# Multi-scale curvature for automated identification of glaciated mountain landscapes^☆^

**DOI:** 10.1016/j.geomorph.2013.11.026

**Published:** 2014-03-15

**Authors:** Günther Prasicek, Jan-Christoph Otto, David R. Montgomery, Lothar Schrott

**Affiliations:** a Department of Geoinformatics – Z_GIS, University of Salzburg, Hellbrunnerstr. 34, 5020 Salzburg, Austria; b Department of Geography and Geology, University of Salzburg, 5020 Salzburg, Austria; c Department of Earth and Space Sciences, University of Washington, Seattle, WA 98195, USA; d Department of Geography, University of Bonn, 53115 Bonn, Germany

**Keywords:** Glaciation, Valley, Morphometry, Curvature, Automation, Multi-scale

## Abstract

Erosion by glacial and fluvial processes shapes mountain landscapes in a long-recognized and characteristic way. Upland valleys incised by fluvial processes typically have a V-shaped cross-section with uniform and moderately steep slopes, whereas glacial valleys tend to have a U-shaped profile with a changing slope gradient. We present a novel regional approach to automatically differentiate between fluvial and glacial mountain landscapes based on the relation of multi-scale curvature and drainage area. Sample catchments are delineated and multiple moving window sizes are used to calculate per-cell curvature over a variety of scales ranging from the vicinity of the flow path at the valley bottom to catchment sections fully including valley sides. Single-scale curvature can take similar values for glaciated and non-glaciated catchments but a comparison of multi-scale curvature leads to different results according to the typical cross-sectional shapes. To adapt these differences for automated classification of mountain landscapes into areas with V- and U-shaped valleys, curvature values are correlated with drainage area and a new and simple morphometric parameter, the Difference of Minimum Curvature (*DMC*), is developed. At three study sites in the western United States the *DMC* thresholds determined from catchment analysis are used to automatically identify 5 × 5 km quadrats of glaciated and non-glaciated landscapes and the distinctions are validated by field-based geological and geomorphological maps. Our results demonstrate that *DMC* is a good predictor of glacial imprint, allowing automated delineation of glacially and fluvially incised mountain landscapes.

## Highlights

• Morphometric analysis was carried out to automatically identify glaciated valleys
• U- and V-shaped valley cross sections were distinguished using multi-scale curvature
• Difference of Minimum Curvature was introduced to describe morphological differences
• The automated approach was tested in three mountain ranges in the western US
• Results show Difference of Minimum Curvature as a good predictor of glacial imprint

## 1 Introduction

The effect of glacial processes on the geometry of mountain landscapes has been studied since the 19th century and large scale features of alpine glaciation like cirques, hanging valleys, and U-shaped valley cross sections have been described and investigated by generations of geologists. The now-conventional interpretation of U-shaped glacial and V-shaped fluvial valleys probably originated in 1872, when Swiss geologist Franz Joseph Kaufmann concluded that round-bottomed valleys owe their form to glacial erosion (Kaufmann, 1872). In North America, Clarance King recognized the cross-sectional U-shape of the upper valleys in the glaciated district of the Uinta Mountains, Utah and the V-shaped profiles below, and attributed these differences to the effect of glacial erosion (King, 1878). William Morris Davis compiled a variety of morphologic attributes of glaciated mountain landscapes and presented hand-drawn illustrations of V-shaped and U-shaped valleys (Davis, 1906). For decades, consensus on this basic distinction between fluvially and glacially carved valleys was primarily based on a plethora of similar qualitative reports, rather than on quantified and measurable attributes.

Quantitative descriptions of valley cross profiles can capture the essence of valley morphology and provide an effective tool to differentiate between valleys formed by different processes (Li et al., 2001). Two principal models are widely used to achieve mathematical approximation of glacial valley transects: a power law adopted by Svensson (1959) and a second-order polynomial first applied by Wheeler (1984). Both approximations show advantages and limitations in depicting valley cross profiles. Power laws have more potential for understanding cross-sectional shape, whereas quadratic equations offer a more robust description (Harbor and Wheeler, 1992; Li et al., 2001).

In geomorphometry, referred to as quantitative land surface analysis based on digital terrain models (Hengl and Reuter, 2009), polynomials are fitted to a regular neighborhood of grid cells (e.g., a kernel of 3 × 3 cells) to calculate land surface parameters (LSPs) like slope and curvature. For curvature calculation, two approaches are widely used. Second order polynomials have been proposed by Evans (1972), and partial fourth order polynomials were adapted by Zevenbergen and Thorne (1987). The lower order approach is incorporated in the geographic information system Landserf for multi-scale LSP calculation (Wood, 1996). Although mathematical approximation of valley cross sections by power laws or polynomials is widely used (Graf, 1970; Doornkamp and King, 1971; Augustinus, 1992; James, 1996; Schrott et al., 2003), to our knowledge, quantification of cross-sectional valley shape has never been done based on mathematical approximation of the three dimensional land surface instead of a two dimensional cross section. The advantages of a three dimensional approach would be automation, spatially continuous instead of discrete results, and the potential for automated mapping of glaciated valleys.

Identifying the location of recent and past glaciated areas has been an integrated part of glaciology since Agassiz (1840), and plays a crucial role in understanding climate variations and landscape evolution. Presence and extent of Pleistocene glaciation have been mapped throughout the globe, but knowledge is still incomplete in some regions (Ehlers and Gibbard, 2004; Ehlers et al., 2011), and consensus remains elusive in others (Gualtieri et al., 2000; Grosswald and Hughes, 2002; Owen et al., 2008). In addition, evidence for glacial remains on Mars is extensively investigated, and controversial, in planetary science (Head et al., 2003, 2010). Although the importance of glacial mapping is undoubted, implications of automated approaches are widely lacking and existing investigations reveal several drawbacks. d'Oleire-Oltmanns et al. (2013) developed a simple semantic model for automated delineation of drumlins and tested their approach in Bavaria. Agreements between mapped and reference landforms were satisfactory, but the study area covered only about 40 km^2^ and did not include large variations in landform development. Sternai et al. (2011) introduced hypsokyrtomes, a specified derivative of hypsometric curves, to identify the regional glacial imprint of mountain ranges. While their results are promising, *a priori* knowledge about an important variable of glaciation, the mean long-term equilibrium line altitude (ELA), is a prerequisite to apply their approach.

Here we present and test a novel method to automatically identify glaciated mountain landscapes based on digital land surface analysis. We exploit the conventional wisdom of U-shaped and V-shaped valleys to gain simple geomorphometric semantics and identify glacial imprint in three mountain ranges across the western United States. Continuous DTMs are segmented into regular quadrangles of identical size, and finally those quadrangles are classified. We first investigate differences in multi-scale curvature of sample catchments revealing well-established fluvial and glacial morphology to define threshold values for differentiation. We then apply these thresholds to the study areas and validate our results using field mapping from prior studies. Our methodology is designed to identify glaciated valleys in a regional manner and to assign fluvially incised valleys and flat terrain to the general class *non-glaciated*.

## 2 Study areas

**Fig. 1:**
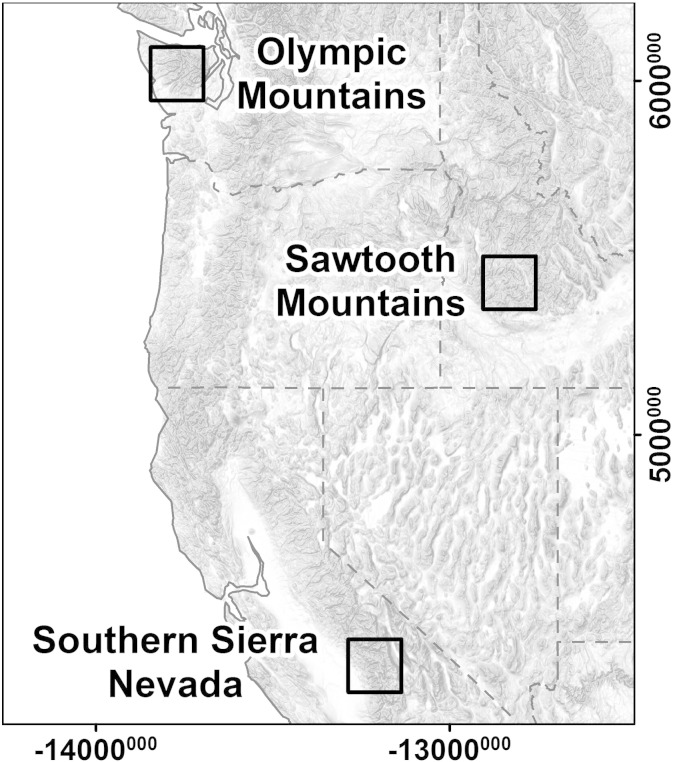
Location of study areas. Spatial reference: WGS84/World Mercator (EPSG 3395).

We test our approach in three study areas in the west of the United States: Sawtooth Mountains, southern Sierra Nevada and Olympic Mountains (Fig. 1). These mountain ranges were selected to test the performance of the approach presented below because of: 1) extensive Pleistocene glaciation; 2) no or very limited recent glaciation; 3) presence of proximal fluvially incised terrain not affected by glaciation; and 4) availability of field mapping of LGM extent or glacial remains for validation.

### 2.1 Sawtooth Mountains and southern Salmon River–Boise Mountains

The Sawtooth Mountains and their western drainages in the southern Salmon River–Boise Mountains area (Fig. 1) primarily consist of Cretaceous biotite granodiorite of the Idaho Batholith and Eocene biotite or hornblende-biotite granite of the Challis magmatic complex. A large block of metamorphic rocks of possible Precambrian age occurs near Stanley Basin (Reid, 1963). Northwest-striking faults of Miocene age and younger caused strong uplift of the rocks underlying the Sawtooth Range. Of these ruptures, only the Sawtooth Fault, an active, range-bounding normal fault on the eastern flank of the Sawtooth Mountains, is known to have had major movement within the last 130 ka (Breckenridge et al., 2003).

Extensive valley glaciers developed in the Sawtooth Range during the Pleistocene, fostered by moist Pacific air masses traversing central Idaho and encountering the mountain barrier (Thackray et al., 2004). Well-developed glacial landforms including deep valley troughs and high jagged peaks are abundant (Reid, 1963; Stanford, 1982; Borgert et al., 1999). However, the western part of the study area has not been affected by glaciers, but shows extensive fluvial relief (Amerson et al., 2008) qualifying for an ideal study site to test our approach. Reconstructed late Pleistocene ELA from Meyer et al. (2004) is used for validation of automated classification results. The ELA rises eastward across the study area from about 2250 to 2650 m. Maps of glacial deposits provide additional validation data (Stanford, 1982; Borgert et al., 1999; Kiilsgard et al., 2001, 2006; Thackray et al., 2004).

### 2.2 Southern Sierra Nevada

The southern Sierra Nevada study area is located in California; about 150 km from the Nevada border (Fig. 1). It extends east–west from Great Basin to Central Valley and from Kings Canyon in the north to Kern Peak in the south. Large sections of the study area belong to Kings Canyon and Sequoia National Park. The bedrock is dominated by granite of Jurassic–late Cretaceous plutons of the Sierra Nevada Batholith (Moore, 1981; Moore and Sisson, 1985). The physiographic history of the area now occupied by the Sierra Nevada remains controversial. Until recently, consensus was that uplift, mainly caused by westward block tilting of the entire range, occurred in several episodes over the last 10 Ma and produced the present elevation only in the Quaternary Period. Alternatively, recent studies argue that the Sierra Nevada was uplifted in the late Mesozoic and remained high or even subsided in the late Cenozoic (Henry, 2009).

The Sierra Nevada was repeatedly glaciated during the climatic fluctuations of the Pleistocene, and Wahrhaftig and Birman (1965) and Clark (1995) mapped the extent of late Pleistocene Tioga glaciers. Here we use subsequent mapping by Gillespie and Zehfuss (2004) for validation of automated identification of glacial mountain landscapes. As in the Sawtooth Mountains study area, glaciation in the southern Sierra Nevada was limited to the higher parts of the range, developing extensive valley glaciers and related morphology. The western drainages retain fluvially-incised morphology with deep canyons, whereas eastern drainages formerly occupied by glaciers descend abruptly to the Great Basin.

### 2.3 Southwestern Olympic Mountains

The study site covers the western, southern and central part of the Olympic Mountains (Fig. 1). We omitted the eastern and northern margins of the range to avoid complex interactions of continental and alpine glaciations. The central Olympic Mountains consist of marine sedimentary rocks of Oligocene to Eocene age, dominated by laminated and/or thin-bedded semischist and slate or phyllite. Miocene lithofeldspatic sandstone and siltstone form part of the western margin of the study area. Southernmost parts of the Olympics are built of the lower–middle Eocene Crescent Formation — tholeiitic basalt flows, basaltic flow breccia, and volcaniclastic conglomerate (Dragovich et al., 2002).

The Olympic Mountains form the first prominent barrier for moist Pacific air in Washington State, were repeatedly affected by alpine glaciations during the Pleistocene, and experience limited recent glaciation concentrated around Mount Olympus and Mount Anderson. Continental ice sheets only affected the northernmost sections of the range, which are not included in our analysis. In contrast to the other study areas, glaciation in the western Olympics was particularly heterogeneous, producing highly variable degrees of glacial imprint in neighboring valleys. Repeated presence of large valley glaciers alternates with no glaciers or smaller glaciers, generally restricted to headwaters (Montgomery, 2002). We used field mapping of Fraser and pre-Fraser glacial moraines (Thackray, 2001; Dragovich et al., 2002) to validate our automated classification results.

## 3 Methods

We developed a method to automatically map glaciated valleys in mountain landscapes based on valley cross-sectional shape. We assumed that valleys with prevailing fluvial imprint typically reveal a V-shaped cross-section with uniformly steep slopes, whereas glacial valleys tend to have a U-shaped profile with a changing slope gradient. Distinction between these two genetic types of valley transects can be performed by analysis of multi-scale curvature to depict valley shape. We adopted this automated land surface analysis approach for a three-dimensional environment and tested the methodology on real-world digital terrain data.

### 3.1 Curvature calculation using fitted polynomials

**Fig. 2:**
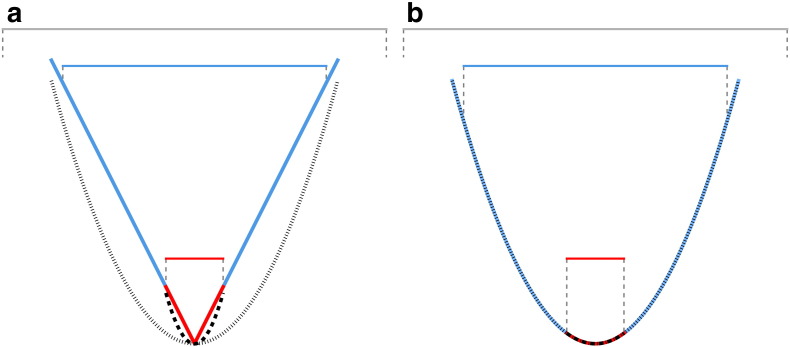
2D scheme of multi-scale curvature analysis: Idealized cross sections of similar sized V-shaped (a) and U-shaped (b) valleys (bold blue) and thalweg subsections (bold red). Horizontal bars and thin, vertical dashed lines indicate valley parts investigated at different scales. Reference scale is marked by red bars, multi-scale valley analysis by blue bars and invalid analysis scale by light gray bars. Dotted lines indicate best-fit second order polynomials for valley cross sections (fine dots) and subsections (bold dots).

Curvature in general indicates to what extent an object is curved. In Fig. 2 the theoretical principles of the methodology are illustrated in 2D for cross-sectional profiles of artificial V-shaped (a) and U-shaped (b) valleys of similar dimensions. Horizontal bars represent different scales of investigation taking into account different parts of the cross sections. To calculate curvature, the following second order polynomial can be fitted to the profile for each scale of investigation (dotted curves in Fig. 2) and curvature is calculated as its second derivative:

(1) $$y=ax^{2}+bx+c\;$$

where *x* is horizontal distance, *y* is height, and a to c are constants.

Note that the ideal glacial valley transect (Fig. 2b) is modeled by a parabola (James, 1996) and therefore is exactly matched by the fitted polynomial. For the ideal V-shaped case (Fig. 2a), the cross-sectional shape is a triangle and the shape of the fitted curve is identical at all scales. On the contrary, the shapes of the polynomials fitted to the U-shaped transect change with scale (Fig. 2b). In Fig. 2a, the curvature of the entire V-shaped cross section (blue V, dotted fitted parabola) is 3.71. The curvature depicting a subsection of the V-shaped graph (red V, boldly dotted fitted parabola) is 3.59. Both curvature values are similar because of the analogy in shape. In contrast, in Fig. 2b, the curvature of the entire U-shaped graph (3.71) is considerably different from the curvature of its subsection (0.74). It has to be emphasized that the fitted polynomials must be normalized according to their extent to achieve similar curvature values for objects of identical shape but different size, as presented in this investigation. Without normalization, curvature would be constant (scale-independent) for U-shaped valleys, whereas it would change with scale of investigation for V-shaped valleys. However, we think that producing similar curvature values for similarly shaped objects independent of size is more intuitive in landform analysis. Therefore, we follow Wood (1996): curvature is calculated in radians per 100 m. To account for differences in object size, the total change is given — a dimensionless ratio providing similar values for similar shapes independent of scale. For example, in Fig. 2 curvature for the entire cross section is obtained in radians per 5000 m, whereas for the subsections it is in radians per 1000 m. This procedure corresponds to resizing the cross sections according to a reference scale.

There are two ways to automatically differentiate between the artificial valley cross sections in Fig. 2: comparison of subsection curvature (red curves), or comparison of the difference of curvature values requiring curvature calculation for at least two scales (red curves and blue curves). The first solution requires high resolution data of a well-defined valley floor. In addition, it will suffer from ambiguity when applied to real world data because of variable size of cross sections and various slope angles resulting from variations in valley sizes and height–width ratios. Therefore, the second option is applied, yielding relative results, allowing for comparison of transects with variable size and height–width ratio, as well as for data of lower resolution.

The two-dimensional concept presented in Fig. 2 can be generalized to three dimensions to investigate a digital land surface, as intended in this work: based on a DTM, curvature is calculated for each cell over a multitude of scales using moving windows of variable width. In geomorphometry, curvature calculated for DTMs can highlight divergent and convergent parts of the landscape and has important implications for surface processes (Carson and Kirkby, 1972). To derive curvature on an irregular digital surface, a polynomial model is commonly interpolated and fitted to the original topography. In addition, a plane intersecting the surface has to be specified because curvature of a three-dimensional object varies with orientation. Curvature can then be calculated from the fully differentiable intersection graph of the fitted polynomial model and the specified plane. Concave features are depicted by negative curvature values in geomorphometry, which is the opposite of the general mathematical convention. Over the last several decades polynomials of varying order and different methods of fitting have been applied to DTMs built of regular grid cells. The most widely used approaches are those of Evans (1979) and Zevenbergen and Thorne (1987), which have been variously adapted by others (Mitášová and Hofierka, 1993; Moore et al., 1993; Shary, 1995). Whatever polynomial model is used, it is fitted to a square neighborhood and centered on a cell in the grid. Evans (1979) suggests fitting the shape of the surface as an interpolated second-order polynomial based on a least squares fit over a 3 × 3 cell moving window:

(2) $$z=ax^{2}+by^{2}+cxy+\mathrm{dx}+\mathrm{ey}+f$$

where a to f are constants.

Zevenbergen and Thorne (1987) exactly fit a partial fourth-order polynomial with nine coefficients through the central cell and its eight neighbors on a rectangular grid:

(3) $$z=ax^{2}y^{2}+bx^{2}y+cxy^{2}+dx^{2}+ey^{2}+f\mathrm{xy}+gx+hy+i$$

where a to i are constants.

The latter method is implemented in ArcGIS. Discussion about advantages and disadvantages of either approach is legion. Schmidt et al. (2003) provide abundant analysis and show that second-order polynomials are more robust, since they are adjusted to the original terrain by least squares fit and therefore perform a considerable amount of smoothing. Partial quartic models are highly sensitive to local variations in input data because they have to exactly meet all input cells. For our multi-scale approach we needed a sound method to interpolate and fit polynomials to varying sizes of neighborhoods. Therefore, we followed Wood (1996) and adopted the robust method of second-order polynomials (Eq. (2)), getting along with six coefficients independent of the number of incorporated grid cells.

Wood (1996) adapted the quadratic parameterization approach of Evans (1979) to perform computation of LSPs on varying neighborhoods for multi-scale analysis, and implemented it in the geographic information system LandSerf, which we used for computation. According to Evans (1979), the six coefficients needed for fitting of a quadratic surface to irregular topography can be derived from six simple equations due to the data arrangement in the regular 3 × 3 cell neighborhood. Conventional least squares fitting is therefore unnecessary. Wood (1996) replaced this simplification by a matrix solution to enable for a neighborhood of up to *n* × *n* cells, only limited by the smaller side of the DTM. In addition, Wood (2009) suggested that a characteristic scale is defined as the scale where an LSP calculated over multiple window widths (neighborhoods) becomes most extreme. We used this concept to perform multi-scale curvature calculation in a three-dimensional environment.

### 3.2 DTM resolution and scales of investigation

The curvature of two scales represented by moving windows (neighborhoods) of regular shape has to be compared to apply this method: a scale depicting mainly the central part of the valley holding the valley floor and a scale including the entire cross section. The first has to be determined by the user and is termed reference concavity scale, because it is identical for all valley sizes. The second varies with cross-sectional valley extent and is termed maximum concavity scale. When analyzing a range of scales, starting with the reference scale, and assuming normalization of the fitted polynomials, maximum concavity scale can be defined by the particular scale leading to most extreme concavity according to Wood (2009). Assuming normalization according to scale, concavity increases with valley depth for a U-shaped valley, hence it shows most concave curvature when most of the valley transect is analyzed. V-shaped valleys can be expected to show similar concavity for all scales of investigation. In either case, the curvature value will become less concave as soon as the scale of investigation gets large enough to overlap with major ridges or neighboring valleys (Fig. 2, light grey bars), and those scales will therefore not be taken into account. This approach automatically adjusts to valley width, only limited by the defined minimum and maximum scales. The minimum scale is the aforementioned reference concavity scale and the maximum scale varies and no upper limit is imposed, up to the width of the study area. Finally two scales are emphasized: the reference concavity scale is user-defined and has the smallest moving window to only take into account central parts of the cross section; the maximum concavity scale is automatically defined by the most concave (most extreme) curvature value of all analyzed scales, following Wood (2009). In this publication, the moving window width (*w*) used to compute reference concavity is termed reference concavity window, and *w* used to compute maximum concavity is called maximum concavity window.

Comparison of the concavity of a valley subsection with the concavity calculated for the whole valley width for valleys of varying scale requires a DTM suitable to resolve narrow valleys and to accordingly define *w* to calculate curvature. Both DTM resolution and *w* can be used to increase or decrease the amount of detail taken into account. Therefore, these variables have to act in concert to provide accurate classification results. Finally, the minimum DTM resolution is determined by reference concavity scale and data points needed. The reference concavity window has to be small enough to not overlap ridges of the narrowest distinctive valleys (including ridges will cause the curvature to take a more convex value, not representing the actual valley) while being wide enough to not overemphasize minor fluvial channels on the floor of glacial valleys. Arbitrary manual measurements carried out across aerial images of all three study areas revealed a minimum cross-sectional width of well-developed valleys of approximately 250 m and a maximum width of upland glacio-fluvial incision of approximately 30 m. Following these findings, we defined a reference concavity scale of 225 m and a DTM resolution of 25 m (approximately 1 arc second on the average latitude of our study areas), allowing for considerable resolution of narrow valleys but smoothing out minor fluvial incisions on glacial valley floors. Consequently, we used the 1 arc second National Elevation Dataset (NED) of the United States.

Detection of the maximum concavity scale requires an adequately defined range of window widths to account for the large variations in valley sizes. For this, we defined a function that increases *w* by a minimum of 2% of the previous window width, but at least by two cells, starting with the reference concavity window. As mentioned above, in theory the maximum of *w* can be any scale large enough to cover the widest valleys. Here it is set to approximately 7 km.

### 3.3 The role of drainage area

In geomorphometry, drainage area is a regional LSP specifying the amount or the area of all grid cells located upstream of a specified cell (Gruber and Peckham, 2009). This parameter can be used to determine cells which carry the line of the lowest points along a valley as a streamline on a DTM, further referred to as *thalweg*. Mainly thalweg cells carry curvature information relevant to our approach, as depicted in Fig. 2. Cells forming ridges or hillslopes are therefore not to be taken into account but have to be ruled out by a drainage area cutoff. This causes only a subset of all grid cells to be left for classification. In order to perform a gapless landscape classification and to reduce the influence of local variations, we aimed to regionalize our results. For this, we followed Sternai et al. (2011) and subdivided the study areas into regular quadrats.

**Fig. 3:**
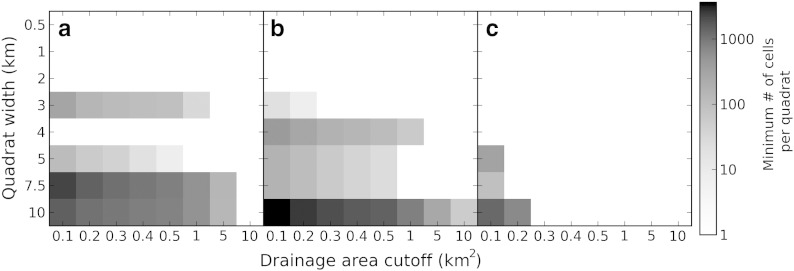
Drainage area cutoff plotted against quadrat width for the Sawtooth Mountains (a), the Sierra Nevada (b), and the Olympic Mountains (c). Minimum amount of valid grid cells per quadrat indicated by logarithmic color map (zero = blank). Partial quadrats are not included.

The drainage area cutoff directly influences the size of the quadrats. A high threshold value (large drainage area) would mainly include thalweg cells of well-developed valleys showing distinct fluvial or glacial morphology most suitable for our approach. But the higher the threshold, the fewer cells are left valid for landscape classification, leading to smaller samples or larger areas for regionalization. Therefore, a tradeoff has to be made between intended unambiguity of morphological differences and amount of grid cells available for classification. To specify the drainage area cutoff for our investigation, we assumed that distinct valley morphology does not occur at drainage areas smaller than the transition zone from divergent to convergent landscapes. This transition has been previously described based on DTMs with a resolution similar to our elevation datasets: Montgomery and Foufoula-Georgiou (1993) showed that transition from debris-flow dominated channels to alluvial channels occurred between 0.1 and 1 km^2^ of the Tennessee Valley, California, indicated by an inflection on slope/area plots. Ijjasz-Vasquez and Bras (1995) recognized a transition zone closely related to those findings, and McNamara et al. (2006) found similar values in a slope/area plot of the Pang Khum Experimental Watershed in northern Thailand. Drainage areas of channel heads mapped in northern Italy by Tarolli and Dalla Fontana (2009) ranged between 0.001 and 0.1 km^2^. All of these studies have been carried out in fluvial morphology, but Brardinoni and Hassan (2006) proposed a threshold between colluvial and fluvial regimes at approximately 0.2 km^2^ for formerly glaciated Coastal British Columbia. These findings indicate that convergent landscapes can be morphometrically identified for grid cells holding an upstream drainage area of ≥ 0.1 km^2^ on DTMs with a resolution of about 1 arc second. Both drainage area cutoff and quadrat size control the amount of pixels per quadrat. To determine suitable values for both parameters we plotted them against each other (Fig. 3) and defined two criteria: 1) each quadrat has to contain grid cells with an upstream drainage area larger than the cutoff, and 2) quadrats should be as small as possible to allow a maximum of detail. Based on the results shown in Fig. 3, and mostly determined by the situation in the Olympic Mountains, the drainage area cutoff was set to 0.1 km^2^ and the quadrat size to 5 × 5 km for this study.

### 3.4 Curvature type

**Fig. 4:**
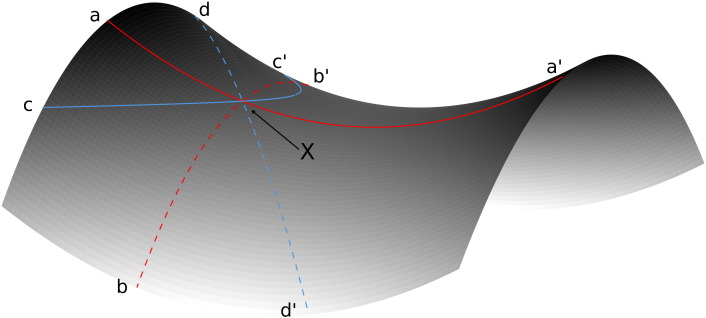
The four directions naturally marked on a surface at point *X*, (after Shary et al. (2002)): a–a′ and b–b′: main normal sections, line of minimum and of maximum curvature, respectively; c–c′: contour line; d–d′: gradient line.

A variety of curvatures, depicting different attributes of terrain shape for different purposes, has been defined in geomorphometry. Shary et al. (2002) state four major directions naturally marked on a surface (Fig. 4), and distinguish between two major types of curvatures: field-specific and field-invariant. The orientation of field-specific curvatures is defined by a vector field (solar irradiation, magnetic, electrical or gravity) and is exemplarily represented by gradient and a contour line in Fig. 4; field-invariant curvatures, also referred to as principal curvatures, are independent of any kind of vector field (line of minimum and maximum curvature in Fig. 4). Our investigation required a curvature type capable of depicting the cross-sectional valley shape for a point on the thalweg. Three major field-specific curvatures fulfill these requirements, dependent on gravity: contour curvature, tangential curvature, and cross-sectional curvature (Schmidt et al., 2003). Besides that, the cross-sectional valley shape is in most cases also represented by the most concave curvature. Therefore, minimum curvature provides relevant information as well. For a point *X* on a surface, minimum curvature is defined as the most concave curvature value given by any normal plane through *X* (Fig. 4). If *X* is located on the thalweg, the minimum curvature intersection is given by a plane perpendicular to the planar direction of flow through the valley. Exceptions to this rule may occur in two cases: poorly developed valleys, and valley parts with profile concavity exceeding cross-sectional concavity. The first exception may only apply to a limited amount of thalweg cells in specific kinds of mountainous areas, like plateaus. The second exception may occur below the drop section of hanging valleys. There, the longitudinal profile of the hanging valley thalweg represents the cross-sectional shape of the main valley, which can be interpreted to be *glacial* or *non-glacial*. Instead, a transect perpendicular to the hanging valley thalweg would follow the flank of the main valley in a more or less parallel direction, carrying no information particularly valuable for our investigation. In this case the behavior of minimum curvature is an advantage over the field-specific curvatures, as below hanging valley drop sections transects parallel or perpendicular to the thalweg can carry the desired information about glacial morphology. Therefore, calculation of minimum curvature is applied for its ability to represent both cross-sectional and profile curvature. Computation is based on the formula provided by Evans (1979) as implemented in the geographic information system LandSerf (Wood, 1996):

(4) $$C_{\min}=‐a‐b‐\sqrt{{\left(a‐b\right)}^{2}+c^{2}}$$

where *C*_min_ is minimum curvature, and a, b, and c are the coefficients derived from fitting of the quadratic surface to irregular topography, common to Eq. (1).

### 3.5 Difference of minimum curvature

To reduce information content per cell from reference concavity and maximum concavity to a single quantity, a simple variable is defined: Difference of Minimum Curvature (*DMC*). It is calculated by subtracting reference concavity from maximum concavity:

(4) $$\mathrm{DMC}=C_{\max}–C_{\mathrm{ref}}$$

where *C*_max_ is maximum concavity being the most concave value over all investigated scales including reference concavity *C*_ref_, which can only take negative values or zero for convergent terrain. In theory, V-shaped cross profiles would lead to a *DMC* value of zero. However, two factors will cause deviation when dealing with real fluvial cross sections: curvature is calculated for a second order polynomial fitted to a variable amount of surface points due to variable scales; and real fluvial cross sections are not perfectly V-shaped and their valley floors show some width. Therefore, an empirically determined *DMC* threshold is needed to identify glacial mountain landscapes. After setting the drainage area cutoff to rule out cells carrying divergent terrain, we adopted probability density functions of remaining valid cells (along drainage lines) in the fluvial and glacial sample areas to specify the *DMC* threshold. It is defined as the intersection point of the two probability density functions and determined separately for all three study areas. In general, a *DMC* value close to zero accounts for fluvial terrain, classified *non-glacial*, and lower (more negative) *DMC* values indicate glacial valleys. Flat areas reveal a similar reference and maximum concavity leading to a *DMC* value close to zero and therefore are classified *non-glacial* as well. Finally, *DMC* is calculated per grid cell and each valid cell (with an upstream drainage area larger than 0.1 km^2^) is classified *glacial* or *non-glacial*. Classes are then assigned to quadrats according to the majority of grid cells. This approach minimizes the influence of extreme values as curvature is strongly dependent on the cross-sectional height–width ratio and a deeper incised valley would automatically lead to higher concavity and lower *DMC* values. To test whether the *DMC* differences of glacial and even deeply incised fluvial valleys are sufficiently large to prevent from ambiguity, we chose study areas comprising fluvial valleys with exceptional relief, including the Kings Canyon in the Sierra Nevada, one of the deepest canyons in North America. However, this dependency on the height–width ratio hinders the calculation of a ‘degree of glaciatedness’ from the continuous DMC values.

## 4 Results

### 4.1 General behavior of multi-scale curvature and DMC

**Fig. 5:**
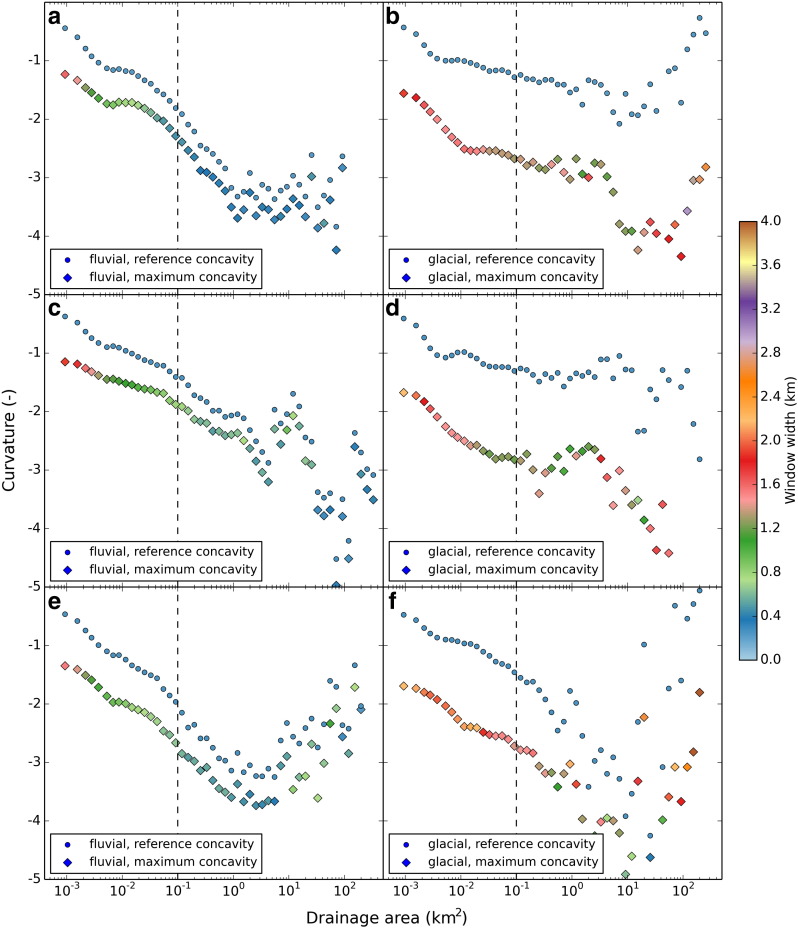
Reference concavity (*C*_ref_), maximum concavity (*C*_max_) and moving window width (*w*) plotted vs. drainage area for the fluvial and glacial sample catchments (displayed in Figs. 10–12) in the Sawtooth Range (a, b), the Sierra Nevada (c, d), and the Olympic Mountains (e, f). Data points are average values calculated per drainage area bin. Bin sizes follow a logarithmic function to control amount of sample cells per bin. Dashed line indicates drainage area cutoff.

**Fig. 6:**
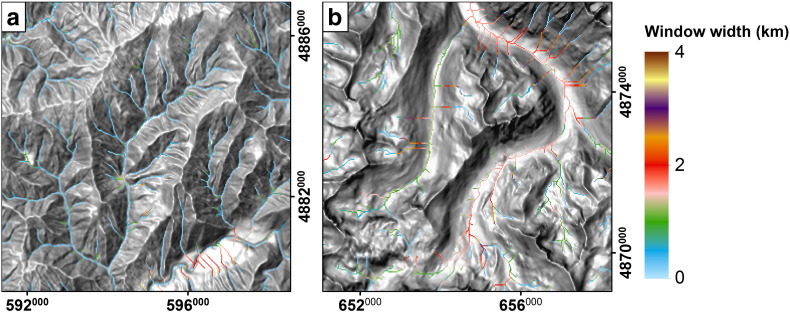
Spatial distribution of moving window width (*w*) for parts of fluvial (a) and glacial (b) sample catchments (displayed in Fig. 10) in the Sawtooth Mountains. Only thalweg cells with drainage area > 0.1 km^2^ are displayed. Spatial reference: WGS84/UTM 11N (EPSG 32611).

We defined fluvial and glacial sample areas with well-documented fluvial or glacial imprint and analyzed the behavior of curvature, *w* and *DMC* over drainage area to determine the drainage area cutoff and the *DMC* threshold. Mean values of *C*_ref_, *C*_max_, and *w* are plotted against drainage area in Fig. 5. Three primary observations emphasizing morphological differences and supporting our hypotheses can be stated: i) *C*_ref_ and *C*_max_ decrease with drainage area for fluvial and glacial sample sites, ii) differences between the two concavity values vary significantly between glacial and fluvial sample catchments, and iii) *w* leading to maximum concavity is considerably larger for glacial than for fluvial sample catchments. This can also be observed per-cell in Fig. 6, where parts of the Sawtooth Mountains are displayed.

**Fig. 7:**
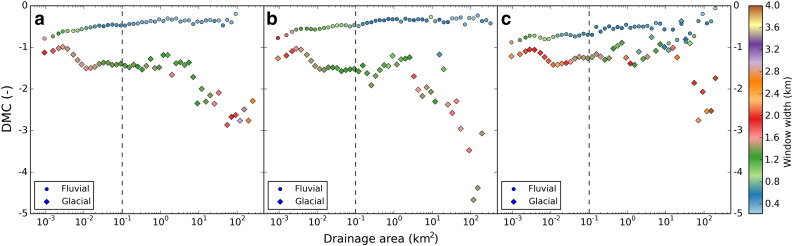
Difference of Minimum Curvature (*DMC*) plotted vs. drainage area for fluvial (circles) and glacial (diamonds) sample catchments (displayed in Figs. 10–12) in the Sawtooth Range (a), the Sierra Nevada (b), and the Olympic Mountains (c). Data points are mean values calculated per drainage area bin. Bin sizes follow a logarithmic function to control amount of sample cells per bin. Dashed line indicates drainage area cutoff.

**Fig. 8:**
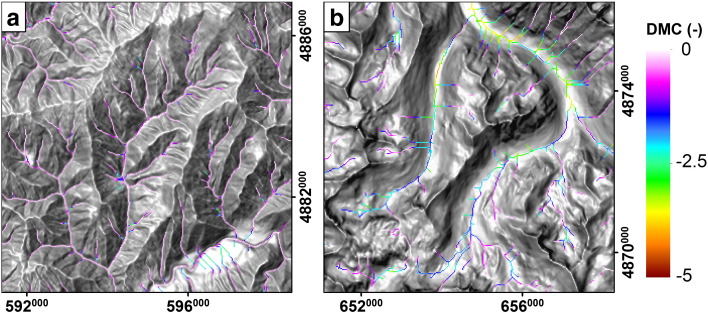
Spatial distribution of Difference of Minimum Curvature (*DMC*) for parts of fluvial (a) and glacial (b) sample catchments in the Sawtooth Mountains (displayed in Fig. 10). Only thalweg cells with drainage area > 0.1 km^2^ are displayed. Spatial reference: WGS84/UTM 11N (EPSG 32611).

**Fig. 9:**
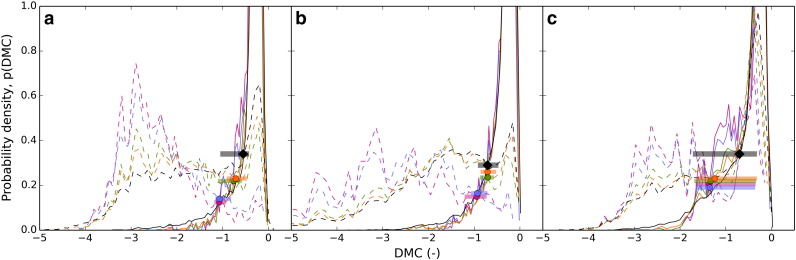
Probability density functions of Difference of Minimum Curvature (*DMC*) for fluvial (continuous) and glacial (dashed) sample catchments (displayed in Figs. 10–12) in the Sawtooth Mountains (a), the Sierra Nevada (b), and the Olympic Mountains (c). Functions are given for drainage area cutoffs of 0.1 (black), 0.5 (orange), 1 (green), 5 (blue), and 10 (purple) km^2^. *DMC* thresholds are marked by bold dots, the threshold applied for classification in this study is indicated with a black diamond.

The behavior of *DMC* for fluvial and glacial sample catchments of the three study areas is illustrated in Fig. 7, showing large glacial–fluvial differences of average *DMC* values. In Fig. 8, *DMC* values are given per cell for cells holding an upstream drainage area > 0.1 km^2^. Fluvial channels mostly reveal a *DMC* close to zero (Fig. 8a), but for glacial areas, *DMC* in general takes large values for valid cells (Fig. 8b). Fig. 9 shows the probability for a cell of being either *glacial* or *non-glacial* for a variety of drainage area cutoffs. Functions for fluvial and glacial sample catchments are considerably skewed. Fluvial *DMC* values reveal a narrow distribution and peak slightly below zero, whereas glacial density functions show a wide range of values. In addition, glacial values reveal a peak slightly below zero, a result of cells showing drainage areas larger than the cutoff combined with low relief. Nevertheless, at a specific *DMC* threshold probabilities switch. A cell holding a *DMC* value smaller than the threshold is more likely to indicate glacial terrain and a cell with a *DMC* value larger than the threshold probably belongs to an area dominated by fluvial incision or low relief. In Fig. 9, *DMC* thresholds for a drainage area cutoff of 0.1 km^2^ are indicated by black diamonds and were determined separately for the three study areas. In addition, error margins are displayed as horizontal bars. They range from the *DMC* value where the probability of a cell being glacial is twice the probability being fluvial (lower margin) to the *DMC* value where the probability of a cell being fluvial is twice the probability being glacial (upper margin).

### 4.2 Sawtooth Mountains and southern Salmon River–Boise Mountains

**Fig. 10:**
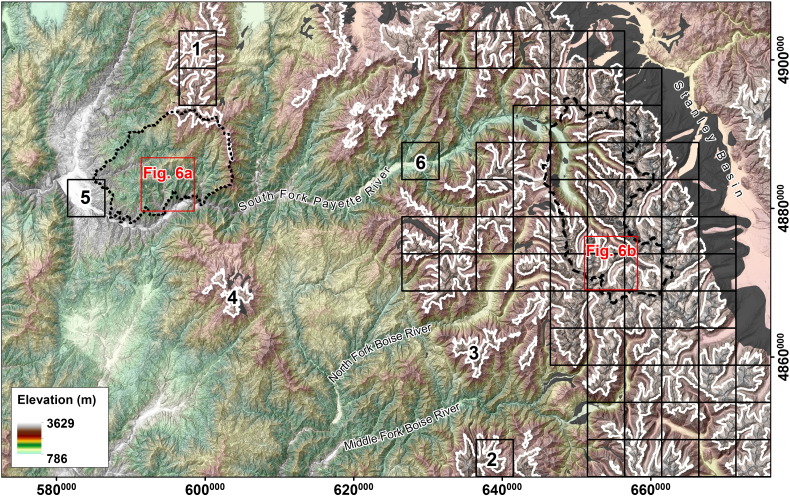
Identification of glacial (black quadrats) mountain regions of the Sawtooth Mountains, Idaho. Sample catchments are indicated by dotted outline (fluvial) and dashed outline (glacial). Red squares mark fluvial and glacial areas shown in Figs. 6 and 8. Field evidence for former glaciation: LGM ELA (white; Meyer et al. (2004) and glacial depositions (dark gray; (Stanford, 1982; Borgert et al., 1999; Kiilsgard et al., 2001, 2006; Thackray et al., 2004). See text for detailed discussion of areas marked with bold letters. Spatial reference: WGS84/UTM 11N (EPSG 32611).

In the Sawtooth Mountains and the southern Salmon River–Bose Mountains, *C*_ref_, *C*_max_, and *w* plotted against drainage area reveal obvious differences between fluvial (Fig. 5a) and glacial (Fig. 5b) sample catchments. In contrast to the fluvial sample catchments, large differences occur between reference and maximum concavity of glacial areas. These variations are reflected in the resulting *DMC* values depicted in Fig. 7a. Probability density functions for fluvial and glacial terrain lead to the definition of a distinct *DMC* threshold of − 0.55 (Fig. 9a, black diamond), which was used to automatically identify glacial mountain landscapes. Results are shown in Fig. 10. The field mapped extents of glacial deposits and the ELA during the last glacial maximum are used to validate the output. The highest elevation portions of the Sawtooth Range with abundant glacial deposits and large areas elevated above the LGM ELA are consistently classified glacial with a sharp and homogenous transition to the Stanley Basin in the East. The western drainages feature several well-developed valleys carved by large valley glaciers, dominated by the South Fork Payette River catchment. In addition, several isolated zones of limited glaciation have been mapped in the study area, not all of them identified as such by the *DMC* threshold criterion. Lightning Ridge (1 in Fig. 10) and Steel Mountain (2) have been glaciated and are classified glacial, and testing based on manually taken cross sections shows distinct development of U-shaped valleys. Areas around Swanholm Peak (3) and Freeman Peak (4) have been glaciated as well, but are classified non-glacial. Manual testing of Swanholm Peak area exhibits four minor U-shaped valleys draining north of the main summit. Freeman Peak shows no well-developed glacial valleys. At the northern border of the study area a considerable fraction of formerly glaciated terrain is not classified accordingly by our algorithm. The area shows less relief than major glaciated parts of the study area and hardly any distinctive U-shaped valleys, a consequence of tectonic activities that also account for the development of the Stanley Basin (Anderson, 1947; Reid, 1963). Garden Valley (5) and a short section of the South Fork Payette River drainage (6), both representing fluvial valleys with wide valley floors, are erroneously classified glacial.

### 4.3 Southern Sierra Nevada

In the Sierra Nevada study area, behavior of *C*_ref_, *C*_max_ and *DMC* for fluvial and glacial sample catchments is similar to the observations in the Sawtooth Range (Figs. 5c,d and 7b). The *DMC* threshold used to identify formerly glaciated parts of the Southern Sierra Nevada is − 0.71 and differs slightly from the value applied to the Sawtooth Range (Fig. 9b).

**Fig. 11:**
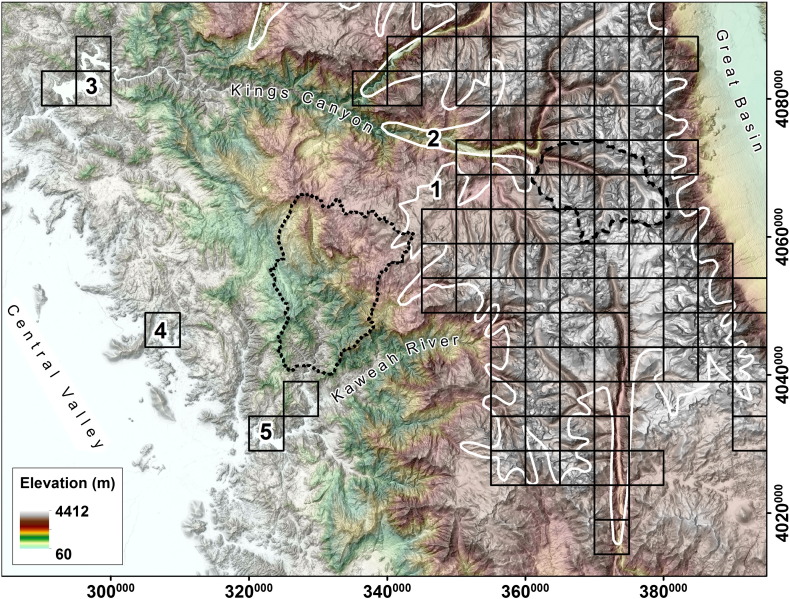
Classification of glacial (black quadrats) mountain regions of the Southern Sierra Nevada, California. Sample catchments are indicated by dotted outline (fluvial) and dashed outline (glacial). Field evidence for former glaciation: glacial extent during LGM (white; Gillespie and Zehfuss (2004). See Results for detailed discussion of areas marked with bold letters. Spatial reference: WGS84/UTM 11N (EPSG 32611).

The extent of the Sierra Nevada LGM used for validation of automated classification results shown in Fig. 11 was published by Gillespie and Zehfuss (2004). In contrast to the Sawtooth Mountains, the study area in the Sierra Nevada shows a very compact distribution of formerly glaciated areas, lacking isolated glaciers on single mountains. The border of this glaciated zone is well depicted by our algorithm and deeply incised fluvial canyons draining the western slopes of the mountain range are classified accordingly. Nevertheless, some formerly glaciated parts of the plateau lack distinct glacial valleys (1 in Fig. 11), leading to misclassification. In the upper drainage of the King's Canyon (2) the maximum extent of the glacier tongue is not correctly represented by automated classification due to non-existent U-shaped valley cross sections. Two lakes in the westernmost fluvial part of the Sierra Nevada study area generate a geomorphometric fingerprint that is mistaken for a glacial valley by our algorithm, Flat Pine Lake (3) and Lake Kaweah (4). Where the Sierra Nevada declines to the Central Valley, another example of a flat floored fluvial valley is mistaken for glacial morphometry (5).

### 4.4 Southwestern Olympic Mountains

Compared with the Sierra Nevada, the Olympic Mountains show a very heterogeneous pattern of glacial imprint, but unlike the Sawtooth Range, no isolated patches of limited past glaciation exist. Due to local climatic variations, valley morphology can change from one valley to the next (Thackray, 2001; Dragovich et al., 2002; Montgomery, 2002), leading to a fringe transition zone between fluvial and glacial mountain areas. In addition, abundant sedimentation affects the morphology of fluvial catchments in the vicinities of former outlet glaciers, and highly-erosive rocks may cause features of former glaciation to be less persistent. This puts our automated classification algorithm to a particular test.

The behavior of *C*_ref_, *C*_max_ and *DMC* differs considerably from the Sawtooth Mountains and the Sierra Nevada (Figs. 5e,f and 7c) causing *DMC* to be less distinctive than in the other study areas. This has implications on the specification of the *DMC* threshold as well (Fig. 9c). It cannot be determined as clearly because of a partially similar progression of the probability density functions. However, the probabilities finally switch at a *DMC* value of − 0.70, similar to the value of the Sierra Nevada.

**Fig. 12:**
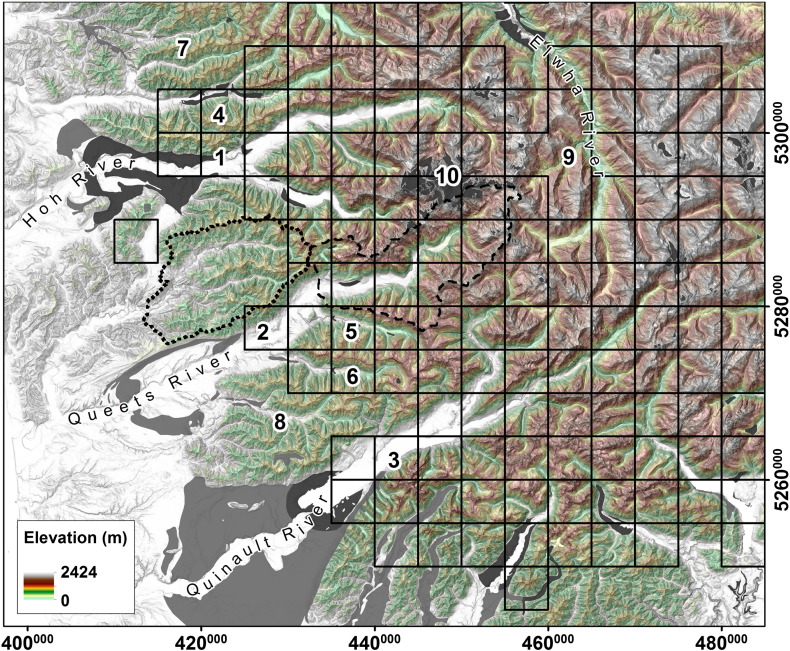
Classification of glacial (black quadrats) mountain regions of the Western Olympic Mountains, WA. Sample catchments are indicated by dotted outline (fluvial) and dashed outline (glacial). Field evidence for former glaciation: deposits of Fraser age (dark gray) and pre-Fraser age (light gray) alpine glaciations (Dragovich et al., 2002). See Results for detailed discussion of areas marked with bold letters. Spatial reference: WGS84/UTM 10N (EPSG 32610).

For validation of the automated classification results (Fig. 12), manual mapping of Quaternary geology is used distinguishing depositions of Fraser and pre-Fraser ages (Dragovich et al., 2002). The formerly glaciated heart of the Olympics is classified accordingly including largest glaciated valleys of Hoh (1 in Fig. 12), Queets (2) and Quinault river (3). However, those valleys are not classified glacial down to the positions of terminal moraines, where relief is low and no distinct glacial valleys are developed. Minor western drainages are identified as *glacial* where Fraser age moraines have developed (4, 5, 6), but are identified *non-glacial* where only pre-Fraser deposits have been found (7, 8). Parts of the Upper Elwha River drainage are classified *non-glacial* as well (9) and manually drawn valley cross sections reveal distinct V-shapes. Detected glacial terrain also includes recent alpine ice (10).

## 5 Discussion

The extent of fluvial and glacial mountain landscapes automatically identified based on the acquired *DMC* thresholds conforms to the field mapping. While the big picture is depicted accurately, some systematic finer-scale misclassification illustrates limitations in our methodology. Fluvial drainages with a large amount of sediment fill are considered to be glacial by our algorithm. Such valleys can be found in all three study areas, and correspond to a limited number of misclassified quadrangles. For further application of this approach, an upper drainage area threshold might therefore be applied. Alternatively, a second indicator could be employed: sediment-filled fluvial valleys may have a *DMC* value similar to glacial ones, but the valley-sides are likely to be steeper for the latter, which can be identified by standard deviation of slope gradient (Evans, 1979). A major reason for abundant sedimentation in fluvial catchments is base level rise, which has been documented in the Sawtooth Mountains (Anderson, 1947; Reid, 1963) with minor but visible impact on our classification. In the Olympic Mountains low-elevation fluvial catchments draining into valleys formerly occupied by large piedmont glaciers show abundant sedimentation as well. Glacial drainages can be considered non-glacial if the relief is very low, leading to a small *DMC* value because of the small curvature values. Such areas in the northern part of the Sawtooth Range study area illustrate the relief dependence of our automated classification approach. Clearly, distinct valleys are needed for differentiation, and both large plateau glaciation areas and broad piedmont moraine spreads cannot be identified.

*w* depicted in Figs. 5 and 7 indicates that cells with very small drainage area reveal maximum concavity at large values of *w*. These cells form ridges or hillslopes, where concavity increases for larger moving windows when parts of adjunct valleys are included in calculation. *w* finally takes values representing valley width at the location of the center cell for drainage areas larger than approximately 0.02–0.5 km^2^ which we believe to mark onset of convergent terrain. The similarity of our assumptions with previous findings (Montgomery and Foufoula-Georgiou, 1993; Ijjasz-Vasquez and Bras, 1995; Brardinoni and Hassan, 2006; McNamara et al., 2006; Tarolli and Dalla Fontana, 2009) suggests plots of characteristic curvature scales against drainage area to be suitable for identification of the hillslope/valley transition over multiple scales.

The drainage area cutoff of 0.1 km^2^ was determined based on the transition zone from divergent to convergent terrain and on considerations about the amount of valid grid cells per quadrat (Section 3.3, Fig. 3). The chosen quadrat size of 5 × 5 km is coherent with the width of the widest valleys in all three study areas and the drainage area cutoff ensures valid grid cells in every quadrat. The probability density plots of *DMC* provided in Fig. 9 show that especially the distribution of glacial *DMC* is greatly affected by the drainage area cutoff. *DMC* values close to zero are typical for fluvial valleys, but exist in glacial terrain as well. In the latter case, they are a result of flat terrain cells holding an upstream drainage area larger than the cutoff. They are less likely if the cutoff is raised, which leads to more negative glacial *DMC*, to larger differences in fluvial and glacial probability density functions, and to variations of the *DMC* thresholds. Although an increase in fluvial-glacial contrast would be favorable for distinction, we did not apply a higher drainage area cutoff because of the associated drop in the number of valid grid cells leading to empty quadrats for regionalization. Using only a slightly higher drainage area cutoff (e.g. 0.2 km^2^), we would have to approximately quadruple quadrat area to avoid empty quadrats. Hence, the number of interpretable grid cells clearly is a governing factor in our methodology.

**Fig. 13:**
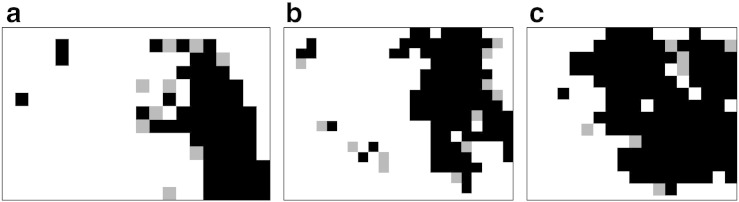
Classification of all three study areas based on a single DMC threshold, the mean (− 0.65) of the three separately defined thresholds. Gray quadrats indicate a change in classification. Sawtooth Mountains (a): change from glacial to fluvial; Sierra Nevada (b) and Olympic Mountains (c): change from fluvial to glacial.

The *DMC* thresholds for differentiation between fluvial and glacial mountain landscapes have been calculated using separate sample catchments for each study area. Nonetheless, threshold values only differ slightly, by about 5% of the *DMC* range. In addition, error margins displayed in Fig. 9 indicate considerable overlap between the *DMC* thresholds determined for the three study areas. The error margins generally decrease with an increase in drainage area cutoff and they show a wide range in the Olympic Mountains — a result of sediment fill within the fluvial sample catchments causing less fluvial–glacial contrast. Full overlap of all three *DMC* thresholds with error margins exists for a drainage area cutoff of 0.1 km^2^. For larger drainage area cutoffs the *DMC* thresholds of the Sawtooth Mountains are still situated within the error margins of the Southern Sierra Nevada and vice versa, but the thresholds of the Olympic Mountains are located outside the error margins of the other two study areas. This is caused by sediment-filled fluvial valleys in the Olympic Mountains and emphasizes the importance of typical fluvial and glacial sample catchments for determination of the *DMC* threshold. Nevertheless, because of minor differences for the 0.1 km^2^ drainage area cutoff, the mean of the three thresholds − 0.65 can be applied to each of the three study areas leading to similar results (Fig. 13). These characteristics of *DMC* can be explained by the principles of the method: V-shaped cross sections theoretically reveal a *DMC* value of 0. However, in reality, fluvial valley sides are not straight and the valley floor has some width. Therefore, in most cases, a *DMC* value slightly below 0 can be expected, which fits our results. *DMC* values of glacial valleys tend to be more negative, causing the *DMC* threshold to mainly depend on the deviation of real fluvial cross sections from the ideal V-shape. Although this deviation may vary due to different influences, for example sediment fill in the fluvial sample catchments of the Olympic Mountains, our classification results account for the general applicability of the V-shape and U-shape concept. Hence, we assume *DMC* thresholds for different mountain ranges to vary in close vicinity of the values we found in the western United States and we expect the existence of a best-fit *DMC* threshold suitable to identify glacial and fluvial imprint in mountain ranges throughout the globe. However, this study presents only a first test of the approach at three study sites and further sampling will be necessary to define such a threshold.

Classification of mountain landscapes into glacial and non-glacial terrain does not account for gradual variation over time leading to intermediate stages and partial overprinting. The Olympic Mountains consist of rocks less resistant to erosion than the other study areas. Therefore we tried to explain our classification results with variations in time since deglaciation and related differences in partial readjustment by splitting validation data into Fraser age and pre-Fraser age remains. Whether these assumptions apply or not, it leads to the general question over which amount of time glacial or fluvial imprint may prevail and builds a bridge to the concept of paraglacial sedimentation (Church and Ryder, 1972). However, gradual changes of glacial landscapes cannot be investigated with the approach presented. As cross-sectional valley shape is strongly dependent on the height–width ratio, a deeper incised valley would inevitably result in higher glaciality. To tackle this problem and to account for ambiguity in the shape of glacial troughs and sediment-filled fluvial valleys, a multi-indicator approach for automated identification of glaciated terrain should be considered for future investigations.

## 6 Conclusion

The conventional geomorphological interpretation of genetic differences in cross sectional valley shapes related to glacial and fluvial erosion constitutes the basis of a novel regional approach quantitatively identifying formerly glaciated mountain landscapes. Concavity values calculated over multiple scales tend to be similar for V-shaped fluvial valleys, but vary over scale for U-shaped glacial valleys. These differences enable automated landscape classification. The approach was tested in three study areas in the western United States yielding promising results. Large areas modified by past glaciation are identified successfully, as are isolated patches of well-developed glacial valleys. Misclassification occurs mostly due to abundant sedimentation leading to wide-floored fluvial valleys and ambiguity in cross sectional valley shape. In general, relief plays an important role in the automated classification routine and distinct valley development is an essential prerequisite. Therefore, this automated approach is restricted to mountain landscapes.

Most mountain ranges around the globe have been investigated by glacial geomorphologists either directly in the field or by remote sensing, but a method like the one presented may be applied to terrain data of Mars and other planetary bodies to investigate the amount of glacial imprint. Further research is underway to combine multiple indicators of glacial terrain for automated detection of more subtle changes in cross-sectional valley morphology, and for calculation of a “glaciality index”.
